# Supplementary material for: Localized Surface Plasmon-Enhanced Infrared-to-Visible Upconversion Devices Induced by Ag Nanoparticles
Source: Materials (Basel). 2023 Feb 28;16(5):1973. doi: 10.3390/ma16051973 (PMC10004485; doi:10.3390/ma16051973)
Supplement: Supplementary file 1 [file materials-16-01973-s001.zip › materials-2216752-supplementary.pdf]

Article

Localized Surface Plasmon-Enhanced Infrared-to-Visible Upconversion Devices Induced by Ag Nanoparticles

Yuyi Zhang [1], Chengjun Liu [1], Xingyu Liu [1], Ziyu Wei [1], Hui Tao [1], Feng Xu [1], Lixi Wang [2], Jiangyong Pan [2], Wei Lei [1,*] and Jing Chen [1,*]

[1] Joint International Research Laboratory of Information Display and Visualization, School of Electronic Science and Engineering, Southeast University, Nanjing 210018, China

[2] School of Electronic and Information Engineering, Nanjing University of Information Science & Technology, Nanjing 210044, China

* Correspondence: lw@seu.edu.cn (W.L.); chenjing@seu.edu.cn (J.C.)

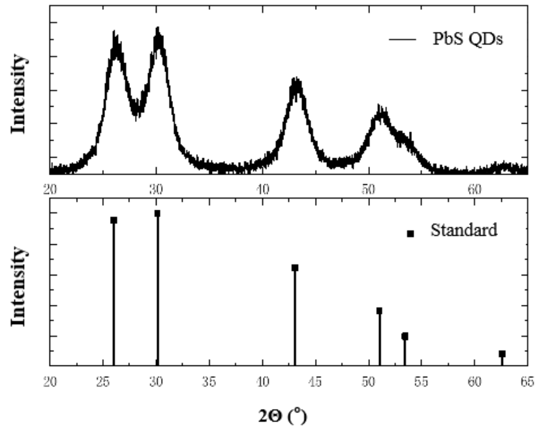

Figure S1. XRD spectra of PbS quantum dots.

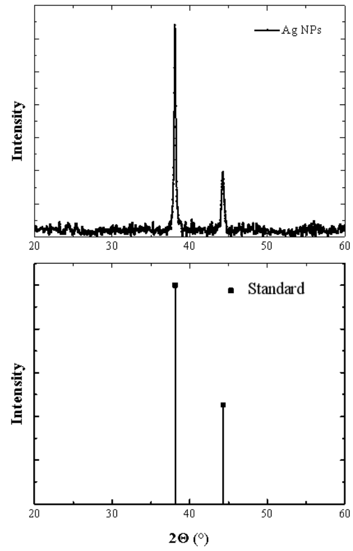

Figure S2. XRD spectra of Ag nanoparticles.

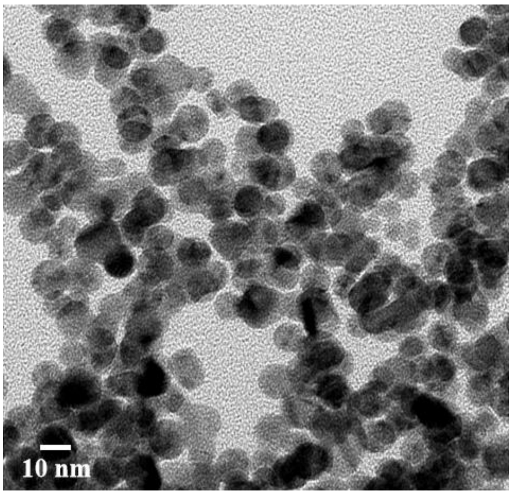

Figure S3. TEM image of Ag nanoparticles.

Table S1. Information about manufacturer and country.

**Manufacturer**	Sigma-Aldrich	Meryer	Sigma	Sinopharm	Aladdin
**Country**	America	China	China	China	China
**Manufacturer**	Macklin	Energy chemical	Maya	Acros	Adamas
**Country**	China	China	China	Belgium	China
